# Ready to deliver maternal and newborn care? Health providers’ perceptions of their work context in rural Mozambique

**DOI:** 10.1080/16549716.2018.1532631

**Published:** 2018-11-02

**Authors:** Sibone Mocumbi, Kevin McKee, Khátia Munguambe, Rogério Chiau, Ulf Högberg, Claudia Hanson, Lars Wallin, Esperança Sevene, Anna Bergström

**Affiliations:** a Department of Obstetrics and Gynaecology, Faculty of Medicine, Universidade Eduardo Mondlane (UEM), Maputo, Mozambique; b Department of Women’s and Children’s Health, Uppsala University, Uppsala, Sweden; c School of Education, Health and Social Studies, Dalarna University, Falun, Sweden; d Department of Public Health, Faculty of Medicine, Universidade Eduardo Mondlane (UEM), Maputo, Mozambique; e Centro de Investigação em Saúde de Manhiça (CISM), Manhiça, Mozambique; f Department of Public Health Sciences, Karolinska Institutet, Stockholm, Sweden; g Department of Disease Control, London School of Hygiene and Tropical Medicine, London, UK; h Department of Neurobiology, Care Sciences and Society, Division of Nursing, Karolinska Institutet, Stockholm, Sweden; i Department of Health and Care Sciences, The Sahlgrenska Academy, University of Gothenburg, Gothenburg, Sweden; j Department of Physiological Science, Clinical Pharmacology, Faculty of Medicine, Universidade Eduardo Mondlane (UEM), Maputo, Mozambique; k Institute for Global Health, University College London, London, UK

**Keywords:** Context assessment, health personnel, maternal and neonatal care, validity, implementation science

## Abstract

**Background**: Deficiencies in the provision of evidence-based obstetric care are common in low-income countries, including Mozambique. Constraints relate to lack of human and financial resources and weak health systems, however limited resources alone do not explain the variance. Understanding the healthcare context ahead of implementing new interventions can inform the choice of strategies to achieve a successful implementation. The Context Assessment for Community Health (COACH) tool was developed to assess modifiable aspects of the healthcare context that theoretically influence the implementation of evidence.

**Objectives**: To investigate the comprehensibility and the internal reliability of COACH and its use to describe the healthcare context as perceived by health providers involved in maternal care in Mozambique.

**Methods**: A response process evaluation was completed with six purposively selected health providers to uncover difficulties in understanding the tool. Internal reliability was tested using Cronbach’s α. Subsequently, a cross-sectional survey using COACH, which contains 49 items assessing eight dimensions, was administered to 175 health providers in 38 health facilities within six districts in Mozambique.

**Results**: The content of COACH was clear and most items were understood. All dimensions were near to or exceeded the commonly accepted standard for satisfactory internal reliability (0.70). Analysis of the survey data indicated that items on all dimensions were rated highly, revealing positive perception of context. Significant differences between districts were found for the *Work culture, Leadership*, and *Informal payment* dimensions. Responses to many items had low variance and were left-skewed.

**Conclusions**: COACH was comprehensible and demonstrated good reliability, although biases may have influenced participants’ responses. The study suggests that COACH has the potential to evaluate the healthcare context to identify shortcomings and enable the tailoring of strategies ahead of implementation. Supplementing the tool with qualitative approaches will provide an in-depth understanding of the healthcare context.

## Background

The inadequate quality of maternal and newborn care is viewed as one of the main reasons for persistent high mortality in low- and middle-income countries (LMIC), including Mozambique [1,2]. The poor quality of care is due not only to constraints on human and financial resources, and weak-functioning health systems [3], but also to the failure to adopt appropriate strategies to implement evidence-based practices (EBPs) [4,5]. Policy-makers must recognize the importance of the healthcare context, not only as an influence on the adoption of appropriate implementation strategies, but also as a modifier of the effectiveness of interventions aimed at increasing health providers’ performance and improving quality of care [6].

The concept of the ‘know-do gap’ has emerged from the field of implementation science, wherein the methods to promote the systemic uptake of research findings and other EBPs in routine healthcare practices are studied, which aims to improve the quality and effectiveness of healthcare [7]. Theoretical frameworks within this field have been developed over the last two decades, often focusing on either the characteristics of the individuals targeted as users of the EBPs to be implemented (and how these characteristics can influence their ability to change routines) or the organization in which these individuals work (and how that organization can cope with change) [8,9]. Common to many of these frameworks is the recognition of the importance of understanding the healthcare context in which evidence is implemented [9]. The Pro-moting Action on Research Implementation in Health Services (PARIHS) framework suggests that successful implementation of evidence occurs as a function of, and the interplay between, characteristics of the evidence to be implemented, the context in which the evidence is implemented and the type of facilitation used to support the implementation [10]. Context is seen as existing on a continuum, from those supporting the use of evidence (high context) moving to those who do not support the use of evidence (low context), and comprises three sub-elements: culture, leadership, and evaluation [10]. Tools aimed at assessing context, and developed within the PARIHS framework, include the Alberta Context Tool (ACT) [11], the Context Assessment Index [12] and the Organizational Readiness to Change Assessment (ORCA) tool [13].

These tools are already being used in several high-income countries, but there has been a lack of appropriate context assessment tools developed for use in LMICs. Consequently, the Context Assessment for Community Health (COACH) tool was developed in Bangladesh, Vietnam, Uganda, South Africa and Nicaragua to assess modifiable aspects of the healthcare context that may influence the implementation of interventions and the integration of EBPs into clinical routines in LMICs [14–16]. The COACH concept originated from the context element of the PARIHS framework [10,17–19] and the interconnected health system building blocks presented by the World Health Organization (WHO) [20]. The tool evaluates health providers’ perceptions of the building blocks of the local health system. COACH has three functions: (1) to enhance opportunities to act on locally identified shortcomings of the health system to increase effectiveness; (2) to guide planning and promote adaptation of implementation strategies in the local context; and (3) to link contextual characteristics to outcome indicators of healthcare interventions. COACH has been found to have psychometrically acceptable properties amongst physicians, nurses, midwives and community health providers in the countries where it was developed [16]. However, being a relatively new tool there is a need to generate further evidence to establish reliability and validity in diverse samples and settings [14].

This article presents findings from a study that used the COACH tool [16] to understand how health providers in Mozambique perceive their work context. Specifically, this study aimed to (1) investigate the comprehensibility and the internal reliability of COACH in a sample of health providers, and (2) to describe the context of maternal healthcare in six districts of Maputo and Gaza provinces in the southern part of Mozambique.

## Methods

### Study design, settings, and participants

**Figure 1.**
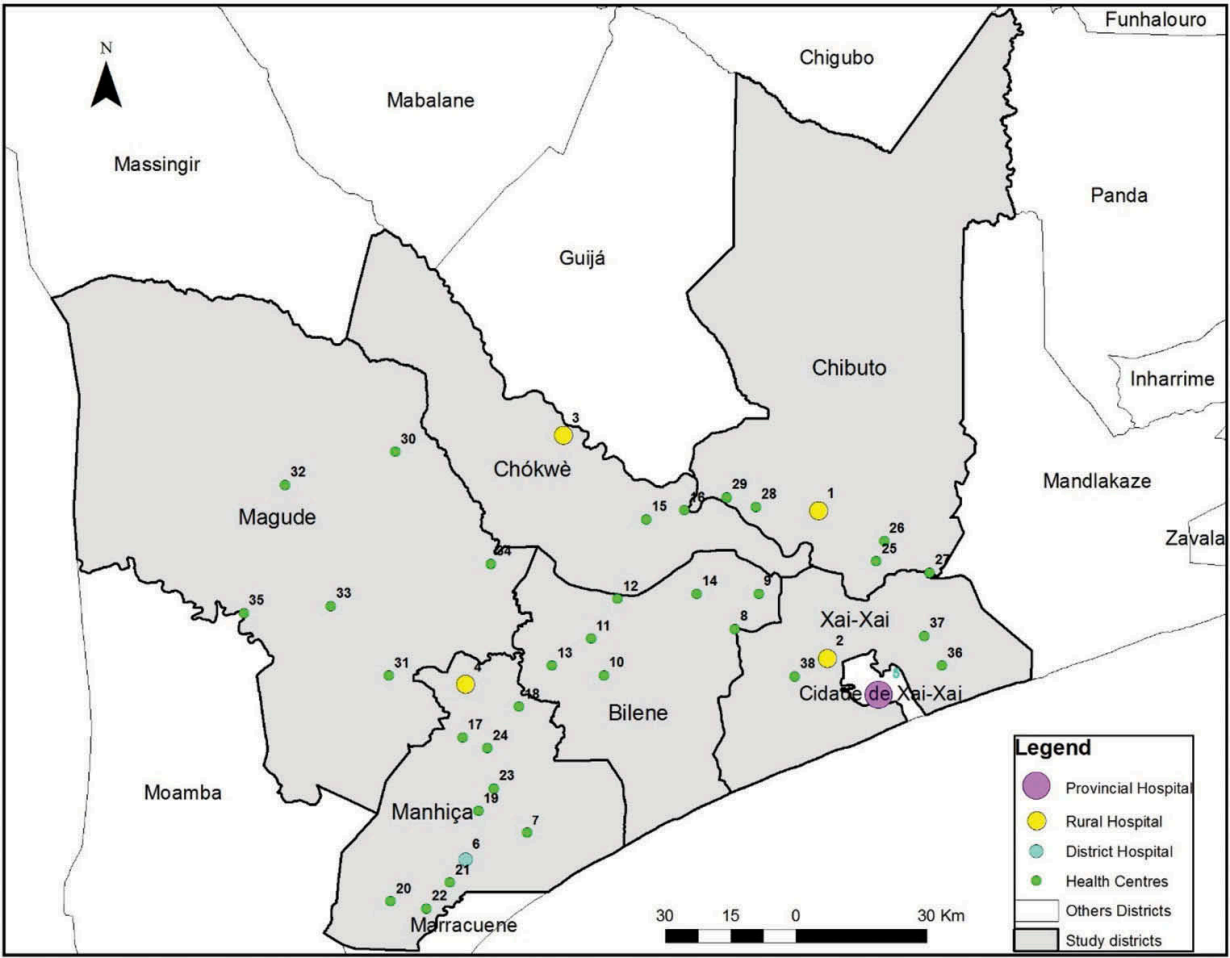
Study setting displaying included districts and health facilities, in Maputo and Gaza provinces, Mozambique.

This was a cross-sectional survey in which the COACH tool was administered to health providers involved in maternal and neonatal care in 38 health facilities of six districts in southern Mozambique (Figure 1): Bilene-Macia, Chibuto, Chokwe and Xai-Xai districts (in Gaza province), and Magude and Manhiça districts (in Maputo province).

Of the 38 facilities, 32 are primary health centres providing essential preventive and curative services, including antenatal and intrapartum care for uncomplicated deliveries. The remaining six facilities are hospitals (four rural, one district and one provincial) to which complicated cases are referred, and routine surgical interventions such as caesarean sections or obstetric hysterectomies, are performed.

### Study tool

The tool has 49 items that measure eight dimensions of context (some dimensions have sub-dimensions) and is available in English, Bangla, Vietnamese, Lusoga, isiXhosa, and Spanish [21]. Items for seven of the eight dimensions (see Table 1 for the definitions) measure agreement with statements that theoretically reflect a context supportive of change (hereinafter referred to as the context’s readiness to change). Items on these dimensions were measured on a five-point Likert scale (ranging from ‘*strongly disagree*’ to ‘*strongly agree*’).

**Table 1.** Definitions of COACH dimensions.

Dimension *Sub-dimensions*	Definition*
**Organizational resources** *Human resources, Space, Communication and transport, Medicines and equipment, Financing*	The availability of resources that allow an organization (unit) to adapt successfully to internal and external pressures
**Community engagement**	The mutual communication, deliberation and activities that occur between community members and an organization (unit)
**Monitoring services for action**	The process of using locally derived data to assess performance and plan how to improve outcomes in an organization (unit)
**Sources of knowledge**	The availability and use of sources of knowledge in an organization (unit) to facilitate best practice
**Commitment to work**	The individual’s identification with and involvement in a particular organization (unit)
**Work culture** *Culture of learning and change, Culture of responsibility*	The way ‘we do things’ in an organization (unit) reflecting a supportive work culture
**Leadership**	The actions of a formal leader in an organization (unit) to influence change and excellence in practice achieved through clarity and engagement
**Informal payment** *Informal payment, Nepotism, Accountability*	Payments or benefits given to individual(s) in an organization (unit), which are made outside the officially accepted arrangements, to acquire an advantage or service

*Unit refers to the department or primary health care centre where the respondent is working.

For the *Sources of knowledge* dimension, respondents indicate for each of five knowledge sources whether the source is available, and, where available, the frequency of its use (*never, rarely, occasionally, frequently* and *almost always*) [14]. In addition to the 49 original COACH items, the version used in this study contained seven demographic questions (age, gender, professional qualification, year professional qualification obtained, health facility, department (if applicable) and years working at the current facility).

#### Translation of the tool

The translation of the COACH tool from English to Portuguese followed Brislin’s model as summarized by Yu et al. (2008) [22]. The translation was conducted in four phases: (1) *Forward translation* (English to Portuguese) by a bilingual professional translator with knowledge of the tool in order to assure appropriate language use; (2) *Review* of the translated tool by a monolingual reviewer with no familiarity of or access to the English version; (3) *Backward translation* (Portuguese to English) by a different bilingual professional translator from the one engaged in step (1); and (4) *Comparison* of the original version and the backward-translated version focusing on conceptual clarity and aimed to ensure an appropriate Portuguese translation of the tool.

#### Response process

Comprehensibility, in this study, refers to the extent to which a statement is easy to understand by the reader. To uncover difficulties in understanding the instructions for completing COACH or items in the tool, the Portuguese version was administered by structured interview to six purposively selected health providers (two physicians, two midwives and two auxiliary nurses) representing the provider categories the main survey would target. In each interview, the first author introduced COACH before the participants were asked to read and state their level of agreement with each of the items in the tool and reflect upon whether they had any difficulty understanding its content. Attention was paid to the participants’ level of understanding and whether they had any challenges in rating their level of agreement with the items. Identified problems were translated into English and categorized in two ways: (a) by the magnitude of their effect on the collected data (prominent vs. minor) [14]; and (b) by Conrad and Blair’s taxonomy [23] (see Table 2). All identified problems were also discussed in relation to the underlying cause of the problem, i.e. relating to the content of the item or the Portuguese translation of the item. Based on the findings from the response process, we produced the final Portuguese version of the COACH tool for data collection (http://www.kbh.uu.se/imch/coach).

**Table 2.** Analysis framework for the COACH tool response process in Mozambique.

**Five types of problems in Conrad and Blair’s taxonomy**	
Lexical problems	difficulties in understanding the meaning of a word or a phrase
Inclusion/exclusion problems	difficulties in determining what to include or exclude in a word used in an item
Temporal problems	difficulties in responding to an item if the scale does not fit
Logical problems	when the item has more than one focus or includes, for example, negations or contradictions
Computational problems	residual types of problems
**Magnitude of the problem’s effect on response data**	
Prominent problems	when the participants did not understand the content of the item or had insufficient information to answer the item
Minor problems	when the participants had to reread the content of the item several times and/or asked for help from interviewers but managed to provide a grounded response

### Data collection

The original COACH tool, designed to be a self-administered questionnaire [16], was amended for administration via an individual structured interview to maximize response and item response rates [24]. An interview guide was designed to ensure that the data collection was standardized and that clear, complete and unambiguous responses to the statements were obtained from the respondents. A member of the research team (R.C.) carried out the interviews, which were undertaken in secluded rooms in the health facilities.

Eligible respondents were health providers (doctors, medical assistants, nurses, midwives and auxiliary nurses) who had worked in the targeted facilities for at least 12 months before the study (*n *= 273). Data were collected between April and June 2016. We were able to interview 175 health providers from the identified 273 eligible respondents (64% response rate). From the 98 who did not participate (46% were nurses and 37% auxiliaries), 55 were absent (vacation, illness leave or not on shift), 42 were not able to answer (busy with patients), and one refused to be interviewed. The non-response rate was higher in hospitals, 41% (47 out of 114) compared to 32% (51 out of 159) in primary facilities.

### Data analysis

The 175 questionnaires were checked for completeness of responses, with no missing responses detected. Data were double-entered in OpenClinica software, version 3.1 [25] and imported into SPSS v. 24 [26] and R software (version 3.3.1) [27] for further analyses. For the demographic variables age, gender, professional category, healthcare level, district and years working in the current facility, mean and standard deviation or median and interquartile range as appropriate were calculated for continuous variables and proportion (%) for categorical variables. Items 42 to 47 described elements of context obstructive to the implementation of interventions and EBPs and scores were therefore reverse scored to be consistent with the connotation of the other items.

Items from the Sources of Knowledge dimension were recoded into 0 (*not available, never* and *rarely*), 0.5 (*occasionally*), and 1 (*frequently* and *always*). The internal consistency reliability of each dimension was tested using Cronbach’s α analyses with item trial removal where indicated. Once satisfactory reliability was demonstrated, items within dimensions were summed, and descriptive analysis (minimum and maximum scores, means and standard deviations) of dimensions was performed.

Subsequently, individual-level data were aggregated within districts and one-way analysis of variance (ANOVA) with the *post hoc* Tukey HSD test was performed for each dimension using the district as the group variable. Level of significance was set at *p *< .05.

## Results

The median age of the participants (Table 3), was 31 years. Nearly two-thirds (66 %) were aged between 21 and 34 years. There were more females (81%) than males. Regarding the professional category, the majority was midwives or nurses (66%), followed by auxiliary nurses (22%). The majority of the respondents worked at the primary level of care (62%), one-third at the secondary level of care (33%) and just 6% at the tertiary level. Most of the participants (58%) had worked for less than 5 years in their current facility, with a median of 3 years (minimum of 1 to maximum of 33 years of service).

**Table 3.** Demographic characteristics of the respondents (n = 175) in Maputo and Gaza provinces, 2016.

Respondents characteristics	*N* = 175	
**Age (years)**	**Median**	IQR*
	31	28–38.5
**Gender**	**Frequency**	**Percent**
Female	141	80.6%
Male	34	19.4%
**Professional category**	**Frequency**	**Percent**
Physician	5	2.8%
Surgeon Officer	4	2.3%
Medical Officer	12	6.9%
Nurse/midwife	115	65.7%
Auxiliaries	39	22.3%
**Respondents by healthcare levels**	**Frequency**	**Percent**
Level I	108	61.7%
Level II	57	32.6%
Level III	10	5.7%
**Respondents by districts**	**Frequency**	**Percent**
Bilene	27	15.4%
Chibuto	25	14.3%
Magude	19	10.9%
Chokwe	19	10.9%
Manhiça	51	29.1%
Xai-Xai	34	19.4%
**Years working in the current facility**	**Median**	**IQR**
	3	1–7

*IQR = Inter Quartile Range

### Response process

Overall, the participants found the COACH tool to be clear and they understood most of the items. We identified problems with 11 of the 49 items (six lexical, four logical and one inclusion/exclusion). Two of the logical problems were categorized as prominent whereas the remaining problems were categorized as minor (Appendix Table A1).

### Internal reliability

All dimensions were near or exceeded the commonly accepted standard for satisfactory internal reliability (0.70) for new scales (α range = 0.64 to 0.91). Two dimensions did not meet this standard. We removed three items (24 to 26) in the *Informal payment* dimension and one item (45) in *Sources of knowledge* dimension to improve internal reliability in these two dimensions. Table 4 displays the minimum and maximum scores and the Cronbach Alpha coefficients for the eight hypothesized context dimensions.

**Table 4.** Internal consistency of the COACH tool in Mozambique, 2016.

Dimension	No items	Score range	α^a^
Organizational resources	11	1–5	.80
Community engagement	5	1–5	.82
Monitoring services for action	5	1–5	.82
Sources of knowledge	5	0–1	.64
*Sources of knowledge, items 24–26 removed*	*2*	*0–1*	.*74*
Commitment to work	3	1–5	.82
Work culture	6	1–5	.73
Leadership	6	1–5	.91
Informal payment	8	1–5	.68
*Informal payment, item 45 removed*	*7*	*1–5*	.*70*

^a^Cronbach Alpha coefficients

Further analyses of the survey data used the original six COACH dimensions with acceptable α and the items remaining in the *Sources of knowledge* and the *Informal payment* dimensions following item removal.

### Rating of work context by dimensions

All dimensions except *Organizational resources* were negative-skewed, with means of above 4 on scales ranging from 1 to 5. The mean of the *Sources of knowledge* dimension was 0.7 on a scale ranging from 0 to 1 (see Table 5). Thus, *Organizational resources* had the lowest mean score (mean = 3.2) of all the dimensions, still indicating an overall agreement that resources were sufficient despite the score below the scale midpoint (3) for the *Space* and *Financing* sub-dimensions (see Appendix Table A2). Over 90% of respondents rated agreement with each of four of the five of the items in the *Community engagement* dimension, implying that they perceived that their facility was in active communication with members of their communities (dimension mean score = 4.3). The mean for the *Monitoring services for action* dimension was 4.2, corresponding to an item average of 89% of respondents agreeing with the items within the dimension. Regarding the *Commitment to work* dimension, the mean score of 4.4 corresponded to an item average of 92% of respondents agreeing with the items within the dimension. In the *Work culture* dimension, an average of 92% of respondents agreed with the items within the dimension (corresponding to a dimension mean score = 4.4), implying that they perceived their context as having a work culture supportive of learning and change (sub-dimension mean score = 4.3) and responsibility (sub-dimension mean score = 4.5). Concerning the *Leadership* dimension, an average of 86% of respondents agreed with the items within the dimension (corresponding to a dimension mean score = 4.2). For the *Informal payment* dimension (mean score = 4.3), an average of 94% of respondents disagreed with items describing high levels of informal payment (*informal payment* sub-dimension, mean score = 4.3, reverse scored), an average of 75% of the respondents disagreed with items describing high levels of nepotism (*nepotism* sub-dimension, mean score = 4.3, reverse scored) and the same proportion (85.7%) of respondents agreed with two items describing how efforts were made by their health facility to, respectively, stop clients from providing informal payment to obtain appropriate healthcare services and stop health workers from asking clients for informal payment (*accountability* sub-dimension, mean score = 4.3). For the *Sources of knowledge* dimension (mean score = 0.7), only 3% of the respondents reported that clinical practice guidelines and other printed material for work were not available. Among the remaining respondents, 58% reported that they use clinical practice guidelines and 62% that they use other printed material for work frequently or almost always.

**Table 5.** Summary of context data for health workers individually and aggregated to district level using the COACH tool in Southern Mozambique, 2016.

				Districts mean (SD)						
Dimensions of context *Sub-dimensions*	Number of items	Scale	Total Sample *(n = 175)* mean (SD)	Bilene	Chibuto	Magude	Chokwe	Manhiça	Xai-Xai	ANOVA *p*
**Organizational Resources**	**11**	**1–5**	**3.2 (1.4)**	**3.0 (1.4)**	**3.2 (1.4)**	**3.2 (1.2)**	**3.0 (1.3)**	**3.3 (1.4)**	**3.4 (1.3)**	**0.171**
*Human resources*	*2*	1–5	3.1 (1.2)	3.0 (1.1)	3.3 (1.0)	3.3 (1.1)	2.1 (1.2)	3.4 (1.2)	3.2 (1.2)	-
*Space*	*1*	1–5	2.7 (1.3)	2.4 (1.0)	1.8 (0.9)	2.7 (0.9)	3.0 (1.2)	2.9 (1.5)	3.1 (1.5)	-
*Communication & transport*	*2*	1–5	3.0 (1.6)	2.6 (1.6)	3.1 (1.6)	2.8 (1.5)	2.6 (1.3)	3.2 (1.6)	3.4 (1.6)	-
*Medicines and equipment*	*4*	1–5	3.9 (1.0)	3.9 (1.1)	4.1 (1.0)	3.7 (0.8)	3.7 (1.1)	4.0 (1.1)	4.1 (1.0)	-
*Financing*	*2*	1–5	2.4 (1.2)	2.0 (1.1)	2.1 (1.2)	2.6 (1.1)	2.8 (1.0)	2.2 (1.1)	2.7 (1.2)	-
**Community engagement**	**5**	**1–5**	**4.3 (1.2)**	**4.5 (0.6)**	**4.4 (0.6)**	**4.3 (0.7)**	**4.1 (0.7)**	**4.3 (0.8)**	**4.3 (0.9)**	**0.263**
**Monitoring services for action**	**5**	**1–5**	**4.2 (0.7)**	**4.2 (0.8)**	**4.4 (0.6)**	**4.0 (0.7)**	**4.2 (0.7)**	**4.3 (0.7)**	**4.2 (0.7)**	**0.428**
**Sources of knowledge**	**2**	**0–1**	**0.7 (0.4)**	**0.7 (0.4)**	**0.7 (0.4)**	**0.7 (0.4)**	**0.9 (0.3)**	**0.7 (0.4)**	**0.6 (0.4)**	**0.141**
**Commitment to work**	**3**	**1–5**	**4.4 (0.1)**	**4.6 (0.7)**	**4.4 (0.8)**	**4.0 (1.0)**	**4.0 (0.9)**	**4.4 (0.9)**	**4.5 (0.5)**	**0.453**
**Work culture**	**6**	**1–5**	**4.4 (0.7)**	**4.4 (0.8)**	**4.5 (0.6)**	**4.2 (0.7)**	**4.1 (0.8)**	**4.5 (0.7)**	**4.4 (0.7)**	0.004*
*Culture of learning and change*	*3*	1–5	4.3 (0.7)	4.2 (1.0)	4.3 (0.7)	4.1 (0.7)	4.1 (0.7)	4.4 (0.8)	4.4 (0.6)	-
*Culture of responsibility*	*3*	1–5	4.5 (0.7)	4.7 (0.6)	4.7 (0.5)	4.2 (0.7)	4.1 (0.8)	4.6 (0.7)	4.5 (0.7)	-
**Leadership**	**6**	**1–5**	**4.2 (0.8)**	**4.3 (0.7)**	**4.1 (0.6)**	**3.8 (0.8)**	**4.0 (0.7)**	**4.1 (1.0)**	**4.4 (0.6)**	0.035*
**Informal Payment**	**7**	**1–5**	**4.5 (1.0)**	**4.7 (0.7)**	**4.7 (0.7)**	**4.4 (1.0)**	**4.2 (1.0)**	**4.4 (1.2)**	**4.5 (1.0)**	0.007*
*Informal payment*^a^	*3*	1–5	4.7 (0.7)	4.9 (0.4)	4.9 (0.4)	4.6 (0.7)	4.4 (1.1)	4.8 (0.8)	4.7 (0.8)	-
*Nepotism*^a^	*2*	1–5	4.3 (1.0)	4.5 (0.9)	4.5 (1.0)	4.4 (1.0)	4.0 (1.2)	4.2 (1.0)	4.1 (1.1)	-
*Accountability*	*2*	1–5	4.3 (1.2)	4.7 (0.8)	4.7 (0.5)	4.0 (1.3)	4.3 (0.6)	3.9 (1.6)	4.5 (1.0)	-

^a^Reversed scores were used for negatively worded items

The results of the analyses examining differences in dimension scores across districts (see Table 5) found significant differences between 3 districts in the *Work culture* (between Chokwe and Chibuto and Chokwe and Manhiça), 2 districts in the *Leadership* (between Xai-Xai and Magude) and 3 districts in the *Informal payment* dimensions (Chokwe and Bilene-Macia districts and Chokwe and Chibuto districts). In all the means comparisons in the *Work culture* and in the *Informal payment* dimension Chokwe district had the lower scores. In the *Leadership* dimension Xai-Xai district had the lower score.

## Discussion

There were two main aspects to the present study. First, we wished to determine the comprehensibility and the internal reliability of COACH in a sample of health providers involved in maternal and neonatal care in six districts in the southern part of Mozambique. Second, we wanted to use COACH to describe dimensions of the healthcare context as perceived by these health providers.

Concerning the first aspect, we interpret the response process findings as indicating that COACH was overall understood as intended and, after a few adaptations, we arrived at a comprehensible Portuguese version of the tool. The internal consistency reliability testing was an important step in ensuring that there was a fit between the dimensions and how the respondents rated their level of agreement. One reason for the low scoring in the *Sources of knowledge* dimension could be the unavailability of internet and e-health/m-health devices, as has previously been reported during the development of the COACH tool [16]. Based on our results we removed four items; three relating to *Sources of knowledge* and one to *Informal payments*, before analysing the context by dimensions.

To our knowledge, this is the first study applying the COACH outside the countries where it was developed. The internal consistency of the COACH tool found in the current study provides evidence of its ability to measure its different dimensions consistently.

Regarding the second aspect, this study revealed that health providers involved in maternal and neonatal care in the study area rated all the dimensions high, although with lower scores in *Organizational resources*, as ‘supportive of change’ when seen in a continuum (six out of seven dimensions using a 5-point scale had a mean > 4).

Further, we found significant differences between districts on the *Leadership, Work culture*, and *Informal payment* dimensions. Although the differences at district level that were identified were a consequence of aggregating individual-level data on health professional’s perceptions on the facility where they worked to the district in which these facilities were situated, it is still important to recognize the tool’s ability to detect differences that might be a consequence of management at a higher level. This could suggest that interventions to support the implementation of EBPs in this current setting should be tailored to strengthen these aspects of the context. It is known that it is essential to tailor interventions according to the decision-making needs of health professionals and the characteristics of the context in which they work [28]. According to Baker et al. (2015), interventions that had been tailored to address identified barriers to change are more likely to improve professional practice compared with either no intervention or the dissemination of guidelines [29].

The low agreement mean score on the *Organizational resources* dimension could indicate the respondents’ perceptions of lesser availability of resources in their units. This finding could be consistent with previous reports of low availability of equipment and supplies in the national healthcare service in Mozambique [30,31]. The unreliability of resources for maternal care has also been observed in previous studies from LMICs, such as Tanzania, Uganda and Nepal [32–34], and this has been recognized as a barrier to implement strategies for the improvement of obstetric care [35]. Furthermore, as reported in Tanzania, inadequately stocked and equipped facilities undermine the ability of the health system to provide optimal maternal care [36]. However, as reported by Leslie et al. [37] in an assessment of health system capacity in Haiti, Kenya, Malawi, Namibia, Rwanda, Senegal, Tanzania, and Uganda, even with structural inputs (amenities, equipment and medications) and adherence to evidence-based guidelines indicating a favourable context, health providers might still provide sub-standard care.

The high agreement mean scores observed in the *Community engagement* and *Monitoring services for action* dimensions could be interpreted either as a result of the implementation of the Mozambican Ministry of Health programme for health facilities to maintain active communication and community empowerment/participation in health promotion [38,39], or as a result of the implementation of monitoring and evaluation activities in all health facilities [40]. Because low salaries were found to be particularly demotivating in several LMICs [41], it was unexpected to find that participants’ responses implied a high *Commitment to work*. Indeed, this could be perceived as commitment and devotion on the part of the health providers in trying to provide empathic and responsive care despite a weak health system with a lack of resources, as has been reported in a previous study in Mozambique [42]. However, the high scores observed in the other dimensions suggest an enabling environment for the provision of maternal and neonatal health care, which is surprising. Several studies in Mozambique and others LMICs have suggested that the working health context is still characterized by maternal and child healthcare providers’ negative attitudes and behavior (such as absenteeism, corruption, poor communication and authoritarian or frightening attitudes) [43,44]. Despite the growing evidence of the practice of informal payments in LMICs [45,46], more than 90% of respondents rated disagreement with all the items in the relevant sub-dimension suggesting they perceived their facility to have low levels of informal payment. Such high levels of statement disagreement raise the concern that a social desirability bias may have been operating here. The high agreement mean score on the *Commitment to work* dimension was also surprising as it has been previously reported that health workers in Mozambique combine their salaried work in the public sector with clinical practice with a fee-for-service private clientele [47,48].

### Strengths and limitations

This is the first study using a comprehensive theory-based assessment of context to describe healthcare context from the health provider’s perspective in Mozambique. The comprehensibility of the COACH tool has been assessed through a response process that provided the identification of problems and their resolution before the tool was administered in our survey. Satisfactory internal reliability was attained for all dimensions after the removal of four items. An experienced interviewer, who was external to the Mozambique health system, was trained to conduct the individual structured interviews. This ensured standardized administration of the COACH tool and resulted in questionnaires with no missing data. We cannot eliminate the possibility of biases, most important the social desirability bias [24] as a cause of the high level of negative-skewness found in responses to many items [49]. The participation rate of 64% could also be a limitation to the study, due to the potential for important differences between respondents and non-respondents.

The observed negative-skewedness and low variation in the data raises a question about the usefulness of tools such as the COACH in this particular setting. Qualitative efforts to understand the healthcare context have previously yielded a more diverse description of context [18,19,32]. One strategy might thus be to use mixed-methods (quantitative and qualitative combined) when aiming to understand context in LMICs.

## Conclusion

New tools need thorough psychometric investigations to ensure reliability and validity. This first assessment of the Portuguese version of the COACH tool has found that the translated version is comprehensible and demonstrates good reliability in describing dimensions of the healthcare context in the study setting. The analysis of ratings of COACH items by health providers involved in maternal and neonatal care in six districts of Maputo and Gaza provinces in Mozambique suggest that their healthcare context is highly supportive of change across all dimensions assessed, except with regard to Organizational resources where our findings indicate a context that is neither clearly supportive nor unsupportive. Significant differences between districts were found on the *Leadership, Work culture*, and *Informal payment* dimensions. This suggests that there might be a rationale for assessing the healthcare context ahead of implementing interventions to enable tailoring of implementation strategies that address any identified shortcomings. The COACH tool has potential as an instrument to evaluate the health care context, although the negative skew in the responses to many items is an issue that remains to be addressed. Using qualitative approaches would be beneficial in order to detect and understand any biases operating when using self-report methods and questionnaires to assess any given health care context. Future research should investigate the association between health providers’ perceptions of the context in which services are delivered and the user’s perspective on the quality of care.

## Paper context

The new Context Assessment for Community Health (COACH) tool, developed to assess modifiable aspects of the healthcare context, needs further investigation to determine its psychometric properties. This study demonstrates the internal reliability of the tool in Mozambique. Using COACH reveals an overall positive perception of context by health providers, but it also raises concerns about potential biases.

## Appendix

**Table A1.** Type and magnitude of problems identified for items of the COACH tool in Mozambique and decisions made, 2016.

Dimension/item	Type and magnitude of problems	Decision
**Organizational resources**		
1. My unit has enough workers with the right training and skills to do everything that needs to be done. PROBLEM: *The item contains two elements – enough staff AND having the right training and skills. Respondent unsure how to rate agreement if, e.g. there are not enough staff but the staff available have adequate training and skills?*	Logical, prominent	Explain to the respondent that both elements of the item must be fulfilled to agree that the resource is available.
4. My unit has access to the transport and fuel that are needed to provide healthcare services. PROBLEM: *The item contains two elements – transport AND fuel. Respondent unsure how to rate agreement if, e.g. a vehicle is available but fuel is not?*	Logical, prominent	Explain to the respondent that both elements of the item must be fulfilled to agree that the resource is available.
7. My unit has enough functional equipment, such as a thermometer and blood pressure cuff, to provide healthcare services. PROBLEM: *The examples provided in the item (i.e. thermometer and blood pressure cuff) could be perceived by a respondent as the only equipment that is requested.*	Inclusion/Exclusion, minor	Give more examples of equipment and clarify in the instructions that the examples should not limit the scope of the different equipment and that the respondent should ask for clarification if not sure about the mean of the examples.
9. If the workload increases, my unit can get additional resources such as medicine and equipment. PROBLEM: *does the statement mean that the additional resources needed, in case of increased workload, must be immediately available?*	Lexical, minor	Explain to the respondent that the meaning is whether there is a system to ensure the resources needed are made available ASAP.
**Community engagement**		
NO Problems		
**Monitoring services for action**		
21. My unit regularly compares its work with national or other guidelines. PROBLEM: ‘*National guidelines’ was translated to ‘normas nacionais’, which was not clearly understood by a respondent.*	Lexical, minor	Provide the other synonyms for ‘guidelines’ in Portuguese, e.g. *guias, protocolos”.*
**Sources of knowledge**		
No Problems		
**Commitment to work**		
No Problems		
**Work culture**		
30. My unit is willing to use new healthcare practices such as the guidelines and recommendations. PROBLEM: *The respondent didn’t understand the meaning of ‘willing to use’ in the item. Does it mean that the unit wants to use new healthcare practices or it is planning to use them?*	Lexical, minor	Explain that the concept in this item is the ‘openness to change’.
33. My unit works for the good of the clients and puts their needs first. PROBLEM: The item contains *two topics – the good of the clients AND taking the clients’ needs first…*	Logical, minor	Explain that taking the clients’ needs first implies somehow the client’s wellbeing.
**Leadership**		
38. The leader actively listens, acknowledges, and then responds to requests and concerns. PROBLEM: *the item contains three actions that the leader is supposed to do – listening, acknowledging and responding to requests and concerns. How should we answer the item if the leader completes one action and not one or two of the others?*	Logical, minor	Explain that all three concepts in the item must be fulfilled to answer positively.
39. The leader effectively resolves any conflict that arises. PROBLEM: *what is meant by ‘effectively resolves’: to resolve in a just way? Or in an appropriate way?*	Lexical, minor	Translate in a meaningful way the idea of ‘effectively resolve’ in Portuguese, in the sense that conflicts are managed in a manner aimed at achieving the desired result.
**Informal payments**		
45. Health workers are sometimes absent from work earning money at other places. PROBLEM: *This is a sensitive question. The statement as it is translated in Portuguese, can be perceived as offensive because most of the health workers in Mozambique have to work in other places because the salary they earn is not enough for their basic needs. Could it be said instead that ‘Sometimes health workers are absent because they have to work in other places’?*	Lexical, minor	Find a meaningful way to say ‘earn money’ in Portuguese, according to the actual context, where the health worker’s salary is not enough to meet their basic needs.
48. Efforts are made to stop clients from providing informal payments to obtain appropriate healthcare services. PROBLEM: *The Portuguese translation is not clear in terms of who is making these efforts.*	Lexical, minor	Clarify in the Portuguese version that the efforts mentioned in the sentence are intended to be completed by the health unit staff.

**Table A2.** Descriptive values of items and dimensions of the COACH tool in Mozambique, 2016.

				Number of ‘disagree’ answers		Number of ‘neutral’ answers		Number of ‘agree’ answers		Total number of respondents
Scaled Dimensions/Items	Range	Mean	Median score	*n*	%	*n*	%	*n*	%	*N*
**Organizational Resources**	**1–5**	**3.2**	4	-	-	-	-	-	-	175
*Human resources*	*1***–***5*	*3.2*	*4*	-	-	-	-	-	-	
1. My unit has enough workers with the right training and skills to do everything that needs to be done	1**–**5	2.8	2	96	54.9%	16	9.1%	63	36.0%	175
2. My unit has enough workers with the adequate training and skills to do their job in the best possible way	1**–**5	3.5	4	46	26.3%	13	7.4%	116	66.3%	175
*Space*	*1***–***5*	*2.7*	*2*	-	-	-	-	-	-	
3. My unit has enough space to provide healthcare services	1**–**5	2.7	2	101	57.7%	13	7.4%	61	34.9%	175
*Communication & transport*	*1***–***5*	*3.0*	*4*	-	-	-	-	-	-	
4. My unit has access to the transport and fuel that are needed to provide healthcare services	1**–**5	2.6	2	96	54.9%	15	8.6%	64	36.6%	175
5. My unit has access to communication tools (e.g. telephones or radios) that are needed to provide healthcare services	1**–**5	3.4	4	54	30.9%	8	4.6%	113	64.6%	175
*Medicines and equipment*	*1***–***5*	*3.9*	*4*	-	-	-	-	-	-	
6. My unit has enough medicine to provide healthcare services	1**–**5	4.1	4	18	10.3%	4	2.3%	153	87.4%	175
7. My unit has enough functional equipment, such a thermometer and blood pressure cuff, to provide healthcare services	1**–**5	4.0	4	27	15.4%	3	1.7%	145	82.9%	175
8. My unit has enough disposable medical equipment, such as syringes, gloves and needles, to provide healthcare services	1**–**5	4.2	4	19	10.9%	0	0.0%	156	89.1%	175
9. If the workload increases, my unit can get additional resources such as medicine and equipment	1**–**5	3.4	4	41	23.4%	31	17.7%	103	58.9%	175
*Financing*	*1***–***5*	*2.4*	*3*	-	-	-	-	-	-	
10. My unit receives money according to an established financial plan	1**–**5	2.6	3	73	41.7%	75	42.9%	27	15.4%	175
11. My unit has money that we can decide how to use	1**–**5	2.1	2	98	56.0%	57	32.6%	20	11.4%	175
**Community engagement**	**1–5**	**4.3**	**4**	-	-	-	-	-	-	
12. In my unit we ask community members what they think about the healthcare services that we provide	1**–**5	4.3	4	5	2.9%	10	5.7%	160	91.4%	175
13. In my unit we listen to what community members think about the healthcare services we provide	1**–**5	4.4	4	2	1.1%	8	4.6%	165	94.3%	175
14. In my unit we have meetings with community members to discuss health matters	1**–**5	4.5	5	3	1.7%	9	5.1%	165	93.2%	177
15. In my unit we encourage community members to contribute to improving the health of the community	1**–**5	4.5	5	5	2.9%	5	2.9%	165	94.3%	175
16. In my unit we encourage other organizations to contribute to improving the health of the community	1**–**5	4.0	4	10	5.7%	19	10.9%	146	83.4%	175
**Monitoring services for action**	**1–5**	**4.2**	**4**	-		-		-		
17. I receive regular updates about my unit’s performance based on information/data collected from our unit	1**–**5	4.1	4	9	5.1%	17	9.7%	149	85.1%	175
18. My unit discusses information/data from our unit in a regular, formal way, such as in regularly scheduled meetings	1**–**5	4.4	5	3	1.7%	8	4.6%	164	93.7%	175
19. My unit regularly uses unit information/data to make plans for improving its healthcare services	1**–**5	4.2	4	4	2.3%	13	7.4%	158	90.3%	175
20. My unit regularly monitors its work by comparing it with the unit’s action plans	1**–**5	4.2	4	6	3.4%	17	9.7%	152	86.9%	175
21. My unit regularly compares its work with national or other guidelines	1**–**5	4.2	4	2	1.1%	17	9.7%	156	89.1%	175
**Commitment to work**	**1–5**	**4.4**	**5**	-		-		-		
27. I am proud to work in this unit	1**–**5	4.4	5	7	4.0%	3	1.7%	165	94.3%	175
28. I am satisfied to work in this unit	1**–**5	4.3	4	9	5.1%	8	4.6%	158	90.3%	175
29. I feel encouraged to do my very best at work	1**–**5	4.4	5	10	5.7%	6	3.4%	159	90.9%	175
**Work culture**	**1–5**	**4.4**	**5**	-		-		-		
*Culture of learning and change*	*1***–***5*	*4.3*	*4*	-	-	-	-	-	-	
30. My unit is willing to use new healthcare practices such as guidelines and recommendations	1**–**5	4.3	4	4	2.3%	12	6.9%	159	90.9%	175
31. My unit helps me to improve and develop my skills	1**–**5	4.1	4	13	7.4%	13	7.4%	149	85.1%	175
32. I am encouraged to seek new information on healthcare practices	1**–**5	4.5	5	2	1.1%	7	4.0%	166	94.9%	175
*Culture of responsibility*	*1***–***5*	*4.5*	*5*	-	-	-	-	-	-	
33. My unit works for the good of the clients and put their needs first	1**–**5	4.5	5	6	3.4%	6	3.4%	163	93.1%	175
34. Members of the unit feel personally responsible for improving healthcare services	1**–**5	4.5	5	2	1.1%	11	6.3%	162	92.6%	175
35. Members of the unit approach clients with respect	1**–**5	4.5	5	1	0.6%	9	5.1%	165	94.3%	175
**Leadership**	**1–5**	**4.2**	**4**	-		-		-		
36. I trust the unit leader	1**–**5	4.2	4	5	2.9%	11	6.3%	159	90.9%	175
37. The leader handles stressful situations calmly	1**–**5	4.2	4	8	4.6%	18	10.3%	149	85.1%	175
38. The leader actively listens, acknowledges and then responds to requests and concerns	1**–**5	4.1	4	7	4.0%	16	9.1%	152	86.9%	175
39. The leader effectively resolves any conflicts that arise	1**–**5	4.0	4	10	5.7%	23	13.1%	142	81.1%	175
40. The leader encourages the introduction of new ideas and practices	1**–**5	4.3	4	5	2.9%	12	6.9%	158	90.3%	175
41. The leader makes things happen	1**–**5	4.1	4	10	5.7%	24	13.7%	141	80.6%	175
**Informal Payment**	**1–5**	**4.5**	**5**	-		-		-		
*Informal payment*	*1***–***5*	*4.8*	*5*	-	-	-	-	-	-	
42. Clients must always give informal payment to health workers to access healthcare services	1**–**5	4.7	5	162	92.6%	4	2.3%	9	5.1%	175
43. Clients are treated more quickly if they make informal payments to health workers	1**–**5	4.8	5	165	94.3%	7	4.0%	3	1.7%	175
44. Medicines or equipment that should be available for free to clients have been sold in my unit	1**–**5	4.8	5	167	95.4%	4	2.3%	4	2.3%	175
*Nepotism*	*1***–***5*	*4.3*	*5*	-	-	-	-	-	-	
46. Health workers in my unit give healthcare services to friends and family first	1**–**5	4.3	5	137	78.3%	26	14.9%	12	6.9%	175
47. Health workers in my unit give jobs or other benefits to friends and family first	1**–**5	4.2	5	126	72.0%	30	17.1%	19	10.9%	175
*Accountability*	*1***–***5*	*4.3*	*5*	-	-	-	-	-	-	
48. Efforts are made to stop clients from providing informal payment to get appropriate healthcare services	1**–**5	4.3	5	20	11.4%	5	2.9%	150	85.7%	175
49. Efforts are made to stop health workers from asking clients for informal payment	1**–**5	4.3	5	17	9.7%	8	4.6%	150	85.7%	175

				Number of ‘not available’, ’never’ and ’rarely’ answers		Number of ‘occasionally’ answers		Number of ‘frequently’ and ‘always’ answers		Total number ofrespondents
Non-scaled dimension/Items	Range	Mean	Median score	***n***	**%**	***n***	**%**	***n***	**%**	***N***
**Sources of knowledge**	**0–5**	**0.7**	**2**	-	-	-	-	-	-	**175**
22. Clinical practice guidelines	0**–**1	0.7	1	45	25.7%	29	16.6%	101	57.7%	175
23. Other printed material for work	0**–**1	0.7	1	35	20.0%	32	18.3%	108	61.7%	175
(a) Scaled items:	1-Strongly disagree, 2-Disagree, 3-Neither disagree, nor agree, 4-Agree, 5-Strongly agree.									
	Scores of the dimensions were calculated using individual mean scores									
(b) Non-scaled items:	0 (not available, never and rarely), 0.5 (occasionally), 1 (frequently and almost always)									
	Scores of this dimensions was calculated as total sum of items									
(c) Recoded into three categories	Disagree: (*Strongly disagree* and *disagree*)									
	Neutral: (*Neither disagree, nor agree*)									
	Agree: (*Agree* and *strongly agree*)									
